# The Prevalence of Thyroid Disorders in Patients With Vitiligo: A Systematic Review and Meta-Analysis

**DOI:** 10.3389/fendo.2018.00803

**Published:** 2019-01-15

**Authors:** Jinping Yuan, Chong Sun, Shibin Jiang, Yansong Lu, Yuhui Zhang, Xing-Hua Gao, Yan Wu, Hong-Duo Chen

**Affiliations:** Key Lab of Immunodermatology, Ministry of Health, Department of Dermatology, The First Hospital of China Medical University, Shenyang, China

**Keywords:** vitiligo, thyroid disorders, prevalence, systematic review, meta-analysis

## Abstract

**Background:** Associations between vitiligo and thyroid disorders have been suggested, However, the prevalence of thyroid disorders in vitiligo vary widely.

**Purpose:** To conduct a systematic review and meta-analysis assessing the prevalence of thyroid disorders in patients with vitiligo.

**Method:** The PubMed, Cochrane Library, EMBASE, CNKI (China National Knowledge Infrastructure), Chongqing VIP database, and Wanfang database from inception to August 2, 2018 were systematically searched. The pooled prevalence and its 95% confidence interval (CI) were calculated.

**Results:** A total of 77 eligible studies were identified and included, published from 1968 to 2018. Six thyroid disorders including subclinical hyperthyroidism, overt hyperthyroidism, subclinical hypothyroidism, overt hypothyroidism, Graves disease, and Hashimoto thyroiditis were described. The numbers of relative studies were 54 in overt hypothyroidism, 50 in overt hyperthyroidism, 25 in subclinical hypothyroidism, 19 in Hashimoto thyroiditis, 16 in Graves disease, and 10 in subclinical hyperthyroidism. The highest prevalence was 0.06 (95% CI: 0.04–0.07) in subclinical hypothyroidism, and the lowest was 0.01 in subclinical hyperthyroidism (95% CI: 0.00–0.01) or Graves disease (95% CI: 0.01–0.02).

**Conclusion:** Six thyroid disorders showed various prevalence in vitiligo. The highest prevalence was in subclinical hypothyroidism, and the lowest was in subclinical hyperthyroidism or Graves disease. Screening vitiligo patients for thyroid disorders seem plausible, in an effort to detect potential thyroid diseases or to assess the risk of future onset.

## Introduction

Vitiligo is characterized by the loss of functional skin and mucosal melanocytes, the estimated prevalence is 0.5–2% (1, 2). Currently, the exact pathogenesis of vitiligo remains obscure. The most accredited hypothesis is the autoimmune theory, being sustained by several epidemiological, clinical, and experimental findings (3–5). These studies indicate that melanocyte defects drive vitiligo pathogenesis by triggering an autoimmune response that leads to melanocyte destruction in susceptible individuals. Patients with vitiligo are more likely to suffer from autoimmune conditions than the general population (6). Several studies have suggested vitiligo is associated with a variety of other autoimmune diseases, including thyroid conditions, alopecia areata, type 1 diabetes mellitus, pernicious anemia, and rheumatoid arthritis. Among these, thyroid disorders are common conditions in vitiligo patients, and a recent study showed one of the most frequently observed autoimmune diseases in autoimmune thyroiditis patients was vitiligo (7, 8). A genetic co-localization between vitiligo and thyroid autoantibodies has also been proposed (9). The British guidelines suggested to check the thyroid function for adult patients with vitiligo, the Dutch guidelines recommend that when patients with vitiligo have clinical symptoms of thyroid disease, thyroid function should be tested (10, 11) Herein, we conducted a systematic review and meta-analysis to explore the prevalence of various kind of thyroid disorders in patients with vitiligo.

## Methods

### Electronic Search

The PubMed, Cochrane Library, EMBASE, CNKI (China National Knowledge Infrastructure), Chongqing VIP database, and Wanfang database were systematically searched with different combinations of key words to identify studies on thyroid disorders in vitiligo. The studies published in the period from inception to August 2, 2018 were identified. The search keywords were [vitiligo] AND [thyroid] with [“prevalence” OR “incidence” OR “epidemiology”]. A manual search was performed by checking the reference lists of key studies and review articles before they were excluded to identify additional studies.

### Inclusion and Exclusion Criteria

Studies were included if they met the following eligibility criteria: (1) provided sufficient information to estimate the prevalence of thyroid disorders in patients with vitiligo; (2) published in either English or Chinese language; (3) had the exact diagnosis of thyroid disorders. The exclusion criteria were duplicate data, irrelevant to vitiligo, review, data mistake, not providing sufficient information. Obscure terms, such as thyroid disfunction, thyroid disease, and autoimmune thyroid disease, or no categorical diagnoses were excluded.

### Data Extraction

Data was extracted from each article using a standardized data-abstraction form, designed in advance. All the potentially relevant papers were reviewed independently by two investigators. Disagreements were resolved through discussion. The following characteristics were extracted: first authors' name, year of publication, country area, number of vitiligo patients, number of different type, or stage of vitiligo patients who have thyroid disorders, number of male and female patients, number, or prevalence of thyroid disorders in vitiligo patients, duration of vitiligo, survey age, adults or children.

### Data Analysis

All statistical analyses were carried out in Stata software (v15.0; Stata Corp, College Station, TX, USA) and a *p* < 0.05 was deemed statistically significant. To explore the prevalence of each thyroid disorder in vitiligo patients, the pooled prevalence and its 95%CI were calculated. Random-effects models were used, if the *p* < 0.05, *I*^2^ > 50%, otherwise, a fixed-effect model was selected (*p* > 0.05, *I*^2^ < 50%). Subgroup analyses based on areas, gender, age, vitiligo type, and vitiligo stage were done to assess sources of heterogeneity. Sensitivity analysis was performed by eliminating individual studies one by one. The effect of publication bias was assessed by Egger's test.

## Results

### Study Flow and Characteristics

**Figure 1:**
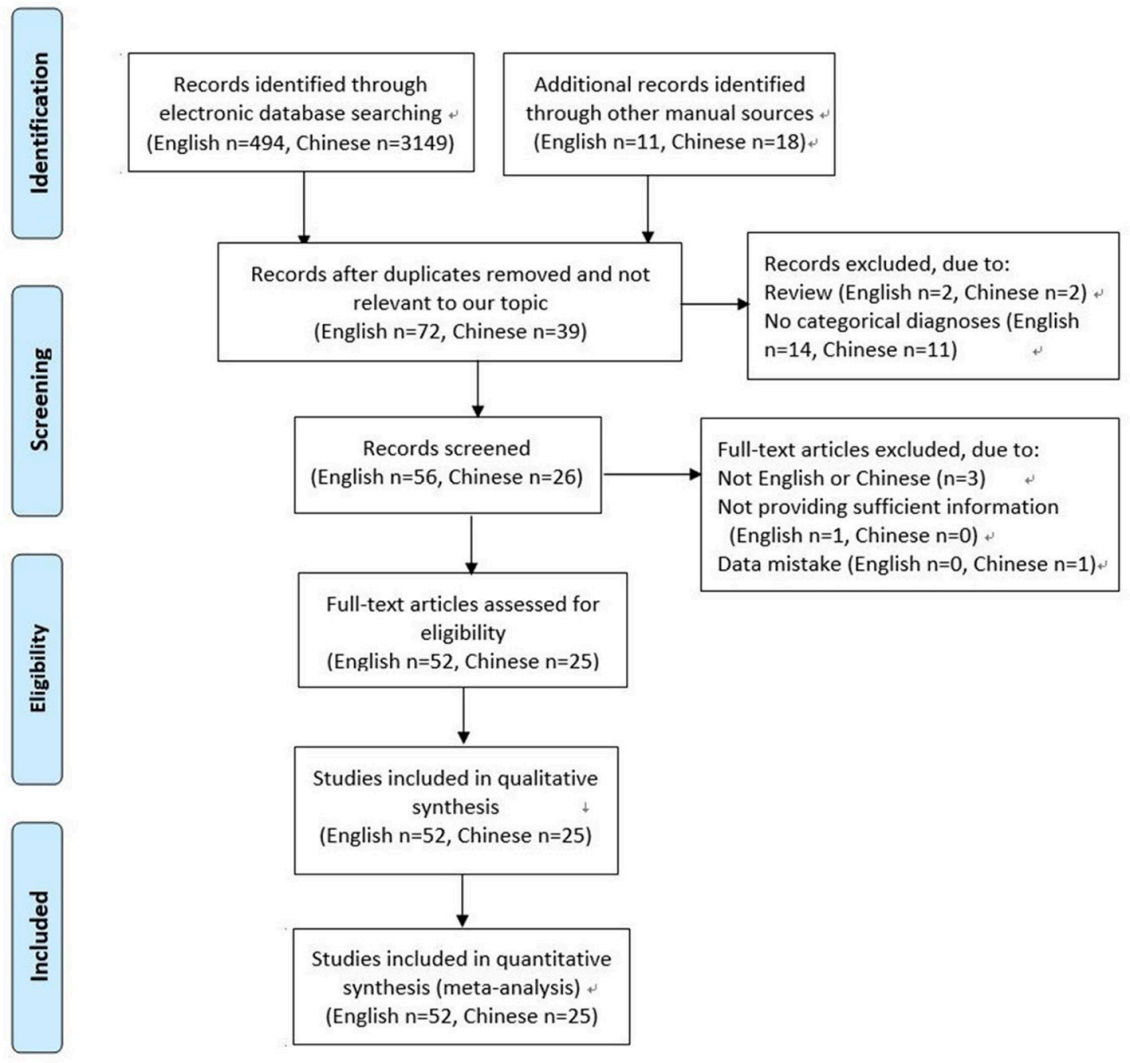
Flow diagram of the study selection process.

A total of 3,643 articles were screened. Of these, 3,566 were excluded for the following reasons: not relevant to our topic, duplication, review, not English or Chinese, not providing sufficient information or data mistake, no categorical diagnoses (for example, thyroid goiter). Finally, 77 studies met the inclusion criteria, and were included in this systematic review and meta-analysis (12–88). Of these studies, 2 studies were reported by one author in the same year, sharing the common basic data, but respectively provided some different data. The detailed selection process was shown in Figure 1.

The characteristics of included studies were described in Table 1. The publication years were from 1968 to 2018. The countries covered France, Netherland, Greece, Serbia, Bulgaria, FRG (the Federal Republic of Germany), Italy, Spain, Austria, England, Denmark, USA, Washington, Colorado, California, Canada, Brazil, China, India, Turkey, Korea, Japan, Iran, Thailand, and Nigeria. The areas covered Europe (France, Netherland, Greece, Serbia, Bulgaria, FRG, Italy, Spain, Austria, England, Denmark), North America (USA, Washington, Colorado, California, Canada), South America (Brazil), Asia (China, India, Turkey, Korea, Japan, Iran, Thailand), and Africa (Nigeria). The number of patients with thyroid disorders ranged from 35 to 73,336.

**Table 1:** Characteristics of studies on the prevalence of thyroid disorders in patients with vitiligo.

**References**	**Country**	**Vitiligo (*n*)**	**Male/Female**	**Duation (years)**	**Survey age (years)**	**Adult/Children**	**Prevalence**					
							**SHyper**	**OHyper (%)**	**SHypo (%)**	**OHypo (%)**	**GD (%)**	**HT (%)**
Wan and Chen (12)	China	324	161/163	—	—	Adult + children	—	3.09	—	—	0.93	—
Vachiramon et al. (13)	Thailand	197	—	—	—	—	—	—	—	—	6.60	4.06
Topal et al. (18)	Turkey	100	51/49	4.9 ± 6.7 (1M−39)	34.9 ± 16.8 (3–78)	Adult + children	—	—	—	5.00	—	—
Yazdanpanah et al. (19)	Iran	72	40/32	—	27.04 ± 1.22	—	—	—	27.78	—	—	—
Kartal et al. (17)	Turkey	155	80/75	—	—	Children	0.65	—	0.65	—	—	1.29
Bae et al. (15)	Korea	73,336	32,519/40,817	—	—	Adult + children	—	—	—	—	0.86	0.75
Wang et al. (16)	China	67	30/37	—	29.15 ± 13.74 (5–70)	Adult + children	—	5.97	—	10.45	—	—
Wang and Wang (14)	China	100	31/69	—	21.6 ± 5.8 (18–62)	Adult	—	17.00	—	—	—	—
Gill et al. (24)	USA	1,098	508/590	—	4.8–99.8	Adult + children	—	0.91	—	8.65	1.09	1.28
Díaz–Angulo et al. (79)	Spain	71	34/37	—	—	Adult + children	1.41	9.86	8.45	1.41	—	—
Chen and Chen (20)	China	352	177/175	55D−4.5	5–56	Adult + children	—	0.57	—	2.27	—	—
Wang et al. (21)	China	60	18/42	—	22 ± 6.4 (18–58)	Adult	—	16.67	—	—	—	—
Cheng et al. (23)	Chia	145	88/57	—	10.73 ± 3.73 (2–17)	Children	—	—	4.14	2.76	—	—
Dash et al. (25)	India	100	41/59	4.64 ± 6.05	29.49 ± 15 (2–62)	Adult + children	—	3.00	—	13.00	—	—
Ma and Li (22)	China	978	540/438	—	37.2 ± 10.7 (5–85)	Adult + children	—	1.33	—	1.64	—	—
Qin (26)	China	413	253/160	—	—	Adult + children	—	0.97	—	—	—	—
Ingordo et al. (33)	Italy	154	52/123	—	—	Adult + children	1.30	3.25	1.95	19.48	—	—
Colucci et al. (31)	Italy	79	26/53	11.67 ± 11.85	38.45 ± 16.0 (18–73)	Adult	2.53	5.06	2.53	3.80	—	—
XU and XU (32)	China	1,386	690/696	—	—	Adult + children	—	5.34	—	2.60	—	—
Wang et al. (30)	China	215	98/117	1W−60	35.14 ± 16.65	Adult	—	3.26	—	0.93	—	1.40
Gopal et al. (28)	India	150	83/67	9–63	3.4 ± 1.77 (3W−26)	Adult + childre	—	0.00	—	20.00	—	—
Zhang et al. (29)	China	60	26/34	12.3 ± 8.2	35 ± 12	Adult	—	—	8.33	—	—	—
Yu and Miao (27)	China	606	309/297	2.96 ± 5.22	23.50 ± 14.79 (2M−74)	Adult + children	—	2.48	—	1.32	—	—
Afsar and Isleten (36)	Turkey	79	29/50	—	8.19 ± 3.45 (2–5)	Children	—	—	16.46	2.53	—	—
Nejad et al. (37)	Iran	86	33/52	6	28.11 ± 12.5	Adult + children	—	6.98	6.98	6.98	—	—
Agarwal et al. (38)	India	268	116/152	1M−10	—	Children	—	2.24	—	6.72	—	—
Sheth et al. (34)	USA	2,441	—	—	—	—	—	1.19	—	7.66	0.98	—
Gey et al. (39)	France	626	216/49	—	31 ± 18.76 (1–74)	Adult + children	1.92	2.56	8.15	8.31	—	—
Kroon et al. (44)	Netherlands	260	110/150	—	—	Children	0.38	—	5.38	1.15	—	—
Yang and Wang (45)	China	540	284/256	23.37 ± 13.45 (1W−42)	—	Adult + children	—	1.30	—	0.74	—	—
Kang et al. (35)	China	521	272/249	—	—	Adult + children	—	1.54	—	—	—	—
Sawicki et al. (43)	Canada	300	141/159	—	41.5 ± 15.5 (11–82)	Adult + children	—	0.67	—	12.00	—	—
Kumar et al. (46)	India	50	21/29	5.5 ± 4.3	42.7 ± 17 (18–70)	Adult	—	0.00	28.00	0.00	—	—
Kroon et al. (47)	Netherlands	434	216/218	—	—	Adult	0.69	1.15	1.61	8.53	—	—
Jian et al. (41)	China	10,000	5,322/4,678	46.17 ± 67.8 (10D−50)	—	—	—	0.52	—	0.14	—	—
Cheng et al. (42)	China	287	143/144	3.0 ± 5.6 (2D−40)	21.8 ± 14.8 (2M−74)	Adult + children	—	0.70	—	1.05	—	—
Wei et al. (40)	China	1,125	573/552	—	—	Children	—	0.09	—	0.18	—	—
Pradhan et al. (48)	India	79	40/39	—	—	Adult + children	—	—	—	1.27	—	—
Nunes and Nunes (50)	Brazil	85	29/56	—	37.14 ± 18.64 (6–78)	Adult + children	—	2.35	1.18	14.12	—	—
Prćić et al. (51)	Serbia	75	28/47	2.6 ± 2.6 (1M−12)	10.81 ± 4.06 (6M−17.7)	Children	—	—	2.66	5.33	—	14.67
Uncu et al. (53)	Turkey	50	26/24	2.26 ± 2.95	9.52 ± 4.54	Children	—	0.00	10.00	0.00	—	—
Narita et al. (54)	Japan	133	57/76	8.2 ± 8.6 (0–63)	49.3 ± 19.8 (3–89)	Adult + children	—	—	—	—	4.51	7.52
Tang et al. (55)	China	1,367	630/737	1M−30	1–79	Adult + children	—	0.29	—	0.15	—	0.07
Poojary (56)	India	204	100/104	—	6M−79	Adult + children	—	—	—	—	0.49	—
Cho et al. (49)	Korea	254	158/166	—	—	Adult + children	0.79	—	2.76		0.79	1.57
Ingordo et al. (52)	Italy	40	40	—	—	Adult	—	2.50	2.50	2.50	—	—
Angulo et al. (57)	Spain	83	39/44	—	36.35 ± 18.83	—	1.40	10.00	—	—	—	—
Akay et al. (59)	Turkey	80	30/50	1M−408M	—	Adult + children	—	2.50	—	1.25	—	31.25
Mazereeuw–Hautier et al. (65)	France	1,14	53/61	—	8.3 ± 0.7 (0.25–15)	Children	—	—	—	9.38	—	—
Paravar and Lee (73)	California	135	55/80	—	2–81	Adult + children	—	2.96	—	14.07	—	—
Altaf et al. (58)	India	192	91/101	—	6–60	Adult + children	—	1.04	12.50	15.10	—	—
Zhou and Fu (61)	China	1,049	462/587	1M−40	18–72	Adult	—	1.81	—	0.57	—	—
Yang et al. (63)	China	363	198/165	1M−11	3–13	Children	—	0.83	—	4.41	—	—
Tanioka et al. (64)	Japan	144	49/49	—	—	—	—	—	—	—	1.39	3.47
Liu et al. (60)	China	1,097	485/612	—	28.8 ± 17.0	Adult + children	—	0.82	—	—	—	—
Zhang et al. (67)	China	6,199	3,276/2,923	1.5 ± 4.5 (0–961M)	24.5 ± 14.6 (1–91)	Adult + children	—	1.16	—	1.00	—	—
Birlea et al. (66)	Colorado	51	18/33	—	49.5 ± 22.8 (2–83)	Adult + children	—	—	—	—	0.00	15.69
Yang and Yang (68)	China	87	43/44	10D−27	32.9 ± 14.3 (4–72)	Adult + children	—	—	1.15	—	1.15	14.94
Sedighe and Gholamhossein (69)	Iran	109	38/79	—	34.41 ± 13 (8–65)	Adult + children	—	—	12.84	14.68	0.92	—
Gopal et al. (74)	India	150	81/69	15D−31	10–55	Adult + children	—	—	—	12.00	—	—
Yang et al. (70)	China	38	13/25	1.5–10	13–56	Adult + children	—	—		2.63	13.16	7.89
Wu et al. (71)	China	3,143	—	—	—	Adult + children	—	0.89	—	0.76	—	—
Fang and Tian (72)	China	562	276/286	2D−43	40D−69	Adult + children	—	2.14	—	0.36	—	—
Daneshpazhooh et al. (75)	Iran	94	48/46	0–40	28.67 ± 15.42	Adult + children	—	—	1.06	—	1.06	—
Laberge et al. (77)	USA	133	—	—	—	—	—	6.02	—	16.54	—	—
Kakourou et al. (76)	Greece	54	23/31	3.7 ± 3.6 (0.16–15.75)	11.4 ± 4.89	Children	—	—	20.47	3.70	—	7.41
Kurtev and Dourmishev (78)	Bulgaria	61	26/35	0.08–11	1.16–16.16	Children	—	5.17	8.62	—	—	—
Iacovelli (81)	Italy	121	40/81	1M−11	3–13	Children	—	0.83	—	4.96	—	—
Onunu and Kubeyinje (80)	Nigeria	351	153/198	—	9M−80	Adult + children	—	0.57	—		—	—
Zettinig et al. (82)	Austria	106	42/64	—	39 ± 18 (6–80)	Adult + children	1.89	—	3.77	11.32	0.00	3.77
Martis et al. (83)	India	100	45/55	—	—	—	—	—	—	2.00	—	—
Hegedus et al. (88)	Denmark	35	—	—	—	—	—	17.14	—	5.71	—	—
Schallreuter et al. (84)	FRG	321	114/207	2M−65	1–85	Adult + children	—	3.74	—	3.43	0.62	0.31
Betterle et al. (85)	Italy	373	138/235	—	7–80	Adult + children	—	—	—	—	4.29	1.88
Grimes et al. (86)	Washington	70	24/46	—	3–73	Adult + children	—	5.71	—	4.29	—	—
Cunliffe et al. (87)	England	56	14/42	—	38 ± 18.6	—	—	5.36	—	—	—	12.50

*SHyper, subclinical hyperthyroidism; OHyper, overt hyperthyroidism; SHypo, subclinical hypothyroidism; OHypo, overt hypothyroidism; GD, Graves disease; HT, Hashimoto thyroiditis; M, month; W, week; D, day*.

Six thyroid disorders were described in the study. They were subclinical hyperthyroidism, overt hyperthyroidism, subclinical hypothyroidism, overt hypothyroidism, Graves disease, and Hashimoto thyroiditis. The number of studies on the 6 above mentioned thyroid disorders in vitiligo patients were 54 on overt hypothyroidism, 50 on overt hyperthyroidism, 25 on subclinical hypothyroidism, 19 on Hashimoto thyroiditis, 16 on Graves disease, and 10 on subclinical hyperthyroidism (Table 2). The data of vitiligo patients who accompanied with one of the following 5 thyroid disorders including thyroid cancer, toxic nodular goiter, thyroid adenoma or asymptomatic atrophic thyroiditis, was not extracted as only 1 study was reported in each disorder.

**Table 2:** The pooled prevalence and subgroup analysis of thyroid disorders in vitiligo patients.

		**Stratified factors**	**No. of studies**	**Prevalence rate**	**Lower limit**	**Upper limit**	**Heterogeneity *I*^**2**^ (%)**	***P* from test of heterogeneity**	**Model**
Subclinical hyperthyroidism		Overall	10	0.01	0.00	0.01	0.0%	0.568	Fixed
	Area	Europe	8	0.01	0.00	0.01	6.2%	0.382	Fixed
		Asia	2	0.01	−0.00	0.02	0.0%	0.869	Fixed
	Gender	Male	2	0.01	−0.00	0.02	100%	—	—
		Female	2	0.01	−0.00	0.01	0.0%	0.795	Fixed
	Age	Children	2	0.00	−0.00	0.01	0.0%	0.719	Fixed
		Adults	2	0.01	0.00	0.02	3.1%	0.310	Fixed
	Type	SV	2	0.00	—	—	—	—	—
		NSV	6	0.01	0.00	0.01	0.0%	0.825	Fixed
	Stage	Active	1	0.02	−0.02	0.07	—	—	—
Overt hyperthyroidism		Overall	50	0.02	0.01	0.02	83.9%	0.000	Random
	Area	Europe	11	0.03	0.02	0.05	65.8%	0.001	Random
		North America	7	0.01	0.01	0.01	49.3%	0.066	Fixed
		South America	1	0.02	−0.01	0.06	—	—	—
		Asia	30	0.01	0.01	0.02	87.7%	0.000	Random
		Africa	1	0.01	−0.00	0.01	—	—	—
	Gender	Male	9	0.01	0.00	0.03	81.6%	0.000	Random
		Female	8	0.02	0.01	0.04	81.9%	0.000	Random
	Age	Children	9	0.01	0.00	0.02	0.702	0.001	Random
		Adults	11	0.05	0.03	0.07	0.864	0.000	Random
	Type	SV	3	0.00	−0.00	0.01	42.3%	0.188	Fixed
		NSV	6	0.06	0.02	0.09	95%	0.000	Random
		Generalized	2	0.04	0.02	0.06	34.7%	0.216	Fixed
		Acrofacial	1	0.00	—	—	—	—	—
	Stage	Active	2	0.05	−0.02	0.11	—	—	—
		Stable	1	0.00	—	—	—	—	—
Subclinical hypothyroidism		Overall	25	0.06	0.04	0.07	83.9%	0.000	Random
	Area	Europe	10	0.05	0.03	0.07	80.3%	0.000	Random
		Asia	13	0.08	0.05	0.11	87.9%	0.000	Random
		North America	1	0.03	−0.02	0.07	—	—	—
		South America	1	0.01	−0.01	0.03	—	—	—
	Gender	Male	4	0.02	0.01	0.03	0.0%	0.521	Fixed
		Female	3	0.03	0.01	0.04	73.6%	0.051	Fixed
	Age	Children	8	0.07	0.03	0.11	85.2%	0.000	Random
		Adults	5	0.05	0.01	0.10	80.4%	0.000	Random
	Type	SV	2	0.00	—	—	—	—	—
		NSV	7	0.04	0.02	0.06	77.5%	0.000	Random
	Stage	Active	2	0.25	0.12	0.38	0.0%	—	—
		Stable	1	0.31	0.15	0.47	—	—	—
Overt hypothyroidism		Overall	54	0.03	0.03	0.04	94.1%	0.000	Random
	Area	Europe	13	0.06	0.04	0.09	85.5%	0.000	Random
		North America	7	0.09	0.07	0.11	74.9%	0.001	Random
		South America	1	0.14	0.07	0.22	—	—	—
		Asia	33	0.01	0.01	0.02	89.8%	0.000	Random
	Gender	Male	10	0.02	0.01	0.03	80.9%	0.000	Random
		Female	9	0.06	0.04	0.08	91.7%	0.000	Random
	Age	Children	10	0.04	0.02	0.06	86.2%	0.000	Random
		Adults	7	0.02	0.01	0.04	86.6%	0.000	Random
	Type	SV	3	0.00	−0.00	0.01	0.0%	0.734	Fixed
		NSV	8	0.03	0.01	0.05	86.8%	0.000	Random
		Generalized	2	0.10	−0.03	0.22	92.6%	0.000	Random
		Acrofacial	1	0.01	−0.00	0.02	—	—	—
	Stage	Active	1	0.02	−0.02	0.07	—	—	—
Graves disease		Overall	16	0.01	0.01	0.02	59.9%	0.002	Random
	Area	Europe	3	0.02	−0.01	0.06	90.4%	0.001	Random
		North America	3	0.01	0.00	0.02	76.1%	0.015	Random
		Asia	10	0.01	0.01	0.02	56.4%	0.014	Random
	Gender	Male	4	0.01	0.01	0.01	58.1%	0.122	Fixed
		Female	4	0.01	0.01	0.01	0.0%	0.502	Fixed
	Type	SV	2	0.00	—	—	—	—	—
		NSV	1	0.01	−0.00	0.02	—	—	—
		Generalized	1	0.02	−0.00	0.04	—	—	—
		Vulgaris	1	0.01	−0.01	0.03	—	—	—
Hashimoto thyroiditis		Overall	19	0.02	0.01	0.03	92.2%	0.000	Random
	Area	Europe	6	0.04	0.01	0.07	83%	0.000	Random
		North America	2	0.08	−0.06	0.22	87.5%	0.005	Random
		Asia	11	0.02	0.01	0.03	94.7%	0.000	Random
	Gender	Male	6	0.00	0.00	0.00	56.8%	0.055	Fixed
		Female	6	0.09	0.04	0.14	85.3%	0.000	Random
	Age	Children	3	0.07	−0.01	0.15	83.9%	0.002	Random
		Adults	1	0.01	−0.00	0.03	—	—	—
	Type	SV	4	0.00	—	—	—	—	—
		NSV	2	0.08	−0.04	0.20	93.6%	0.000	Random
		Generalized	3	0.09	0.06	0.13	20.7%	0.283	Fixed
		Vulgaris	1	0.03	0.00	0.06	—	—	—
		Acrofacial	1	0.10	−0.01	0.21	—	—	—

The diagnoses of subclinical hyperthyroidism were based on the presence of a low TSH level with both normal FT3 value and normal FT4 value and the diagnosis of overt hyperthyroidism was based on the presence of a low TSH level with both raised FT3 value and raised FT4 value (52, 82). The diagnosis of overt hypothyroidism required low FT3 and FT4 values no matter what the TSH level was. Subclinical hypothyroidism was diagnosed on the basis of a raised TSH level with normal T3 and T4 values. Hashimoto's thyroiditis was diagnosed based on the demonstration of circulating thyroid antibodies and diffuse thyroid enlargement or reduced echogenicity on thyroid ultrasonography. And the diagnosis of Graves' disease relies on persistent hyperthyroidism together with positive thyroid antibody and/or increase vascularization on thyroid sonogram, thyroid-stimulating antibodies and diffuse hypercaptation at scintigraphy. Thyroid ophthalmopathy and/or dermopathy are characteristic features of Graves' disease (13).

### Pooled Result of the Prevalence of Thyroid Disorders in Patients With Vitiligo

**Figure 2:**
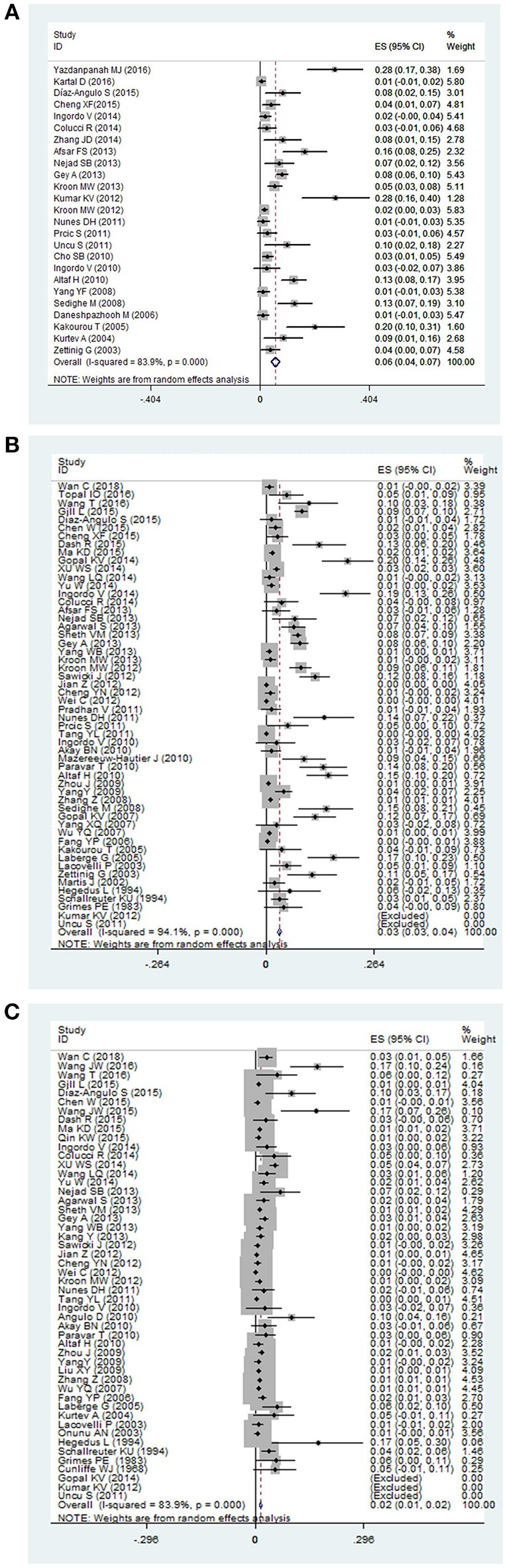
The forest plot of three thyroid disorders in vitiligo patients. The highest prevalence was reported in **(A)** subclinical hypothyroidism, and a majority of investigators paid attention to **(B)** overt hypothyroidism, and **(C)** overt hyperthyroidism in vitiligo patients.

The pooled prevalence of thyroid disorders in patients with vitiligo were showed in Table 2. The highest prevalence of thyroid disorder accompanying vitiligo was 0.06 (95% CI: 0.04–0.07) for subclinical hypothyroidism (Figure 2A). The lowest prevalence was 0.01 (95% CI: 0.00–0.01) for subclinical hyperthyroidism and 0.01 (95% CI: 0.01–0.02) for Graves disease.

### Subgroup Analysis of the Prevalence of Each Thyroid Disorder in Patients With Vitiligo

Potentially distorting factors, including area, vitiligo type, the stage of vitiligo, gender, and age were investigated for subgroup analysis. The areas covered Europe, North America, South America, Asia, Africa. For vitiligo type, segmental vitiligo (SV), non-segmental vitiligo (NSV), generalized vitiligo, acrofacial vitiligo, vulgaris vitiligo were classified. When stratified by the stage, it was divided into active vitiligo and stable vitiligo. For the gender, it was divided into male and female. When stratified by age, the groups were children (<18 years) and adults (≥18 years). The results of subgroup analysis were listed in Table 2.

Overt hypothyroidism in vitiligo patients was reported in 54 studies. The pooled prevalence was 0.03 (95% CI: 0.03–0.04) (Figure 2B). The prevalence in Europe, North America, South America and Asia were found to be 0.06 (95% CI: 0.04–0.09), 0.09 (95% CI: 0.07–0.11), 0.14 (95% CI: 0.07–0.22), and 0.01 (95% CI: 0.01–0.02), respectively. The highest prevalence was 0.14 (95% CI: 0.07–0.22) in South America. Male and female subgroups were 0.02 (95% CI: 0.01–0.03) and 0.06 (95% CI: 0.04–0.08), respectively. The prevalence of overt hypothyroidism in the male population was lower than in females. When stratified by age, the prevalence was higher in children 0.04 (95% CI: 0.02–0.06) than adults 0.02 (95% CI: 0.01–0.04). Pooled prevalence of segmental vitiligo, non-segmental vitiligo, generalized vitiligo, and acrofacial vitiligo were 0.00 (95% CI: −0.00 to 0.01), 0.03 (95% CI: 0.01 to 0.05), 0.10 (95% CI: −0.03 to 0.22), and 0.01 (95% CI: −0.00 to 0.02), respectively. The prevalence in generalized vitiligo was the highest.

Overt hyperthyroidism in vitiligo patients was reported in 50 studies. The pooled prevalence was 0.02 (95% CI: 0.01–0.02) (Figure 2C). The prevalence in Europe, North America, South America, Asia and Africa were found to be 0.03 (95% CI: 0.02 to 0.05), 0.01 (95% CI: 0.01 to 0.01), 0.02 (95% CI: −0.01 to 0.06) and 0.01 (95% CI: 0.01 to 0.02), 0.01 (95% CI: −0.00 to 0.01), respectively. The pooled prevalence in Europe was the highest. Male and female subgroups were 0.01 (95% CI: 0.00–0.03) and 0.02 (95% CI: 0.01–0.04), respectively. When stratified by age, the prevalence was higher in adults 0.05 (95% CI: 0.03–0.07) than children 0.01 (95% CI: 0.00–0.02). Pooled prevalence of segmental vitiligo, non-segmental vitiligo, generalized vitiligo, and acrofacial vitiligo were 0.00 (95% CI: −0.00 to 0.01), 0.06 (95% CI: 0.02 to 0.09), and 0.04 (95% CI: 0.02 to 0.06), respectively. The prevalence of non-segmental vitiligo was higher than the other vitiligo types.

The subgroup analysis of other 4 thyroid disorders including subclinical hyperthyroidism, subclinical hypothyroidism, Graves disease, Hashimoto thyroiditis in vitiligo patients is reported in Table 2.

### Sensitivity Analysis

To examine the stability of the pooled prevalence of thyroid disorders in vitiligo, each study was sequentially excluded for sensitivity analysis. The results demonstrated that some individual studies significantly affected the pooled results in overt hypothyroidism and Hashimoto thyroiditis. The studies of Jian et al. (41) influenced the original results of overt hypothyroidism in vitiligo patients. After removing the study, the pooled prevalence increased by 0.54% (from 3.23 to 3.77%). After removing the study of Bae et al. (15) and Tang et al. (55) of Hashimoto thyroiditis in vitiligo patients, the pooled prevalence increased by 3.47% (from 1.94 to 5.41%).

### Publication Bias

**Figure 3:**
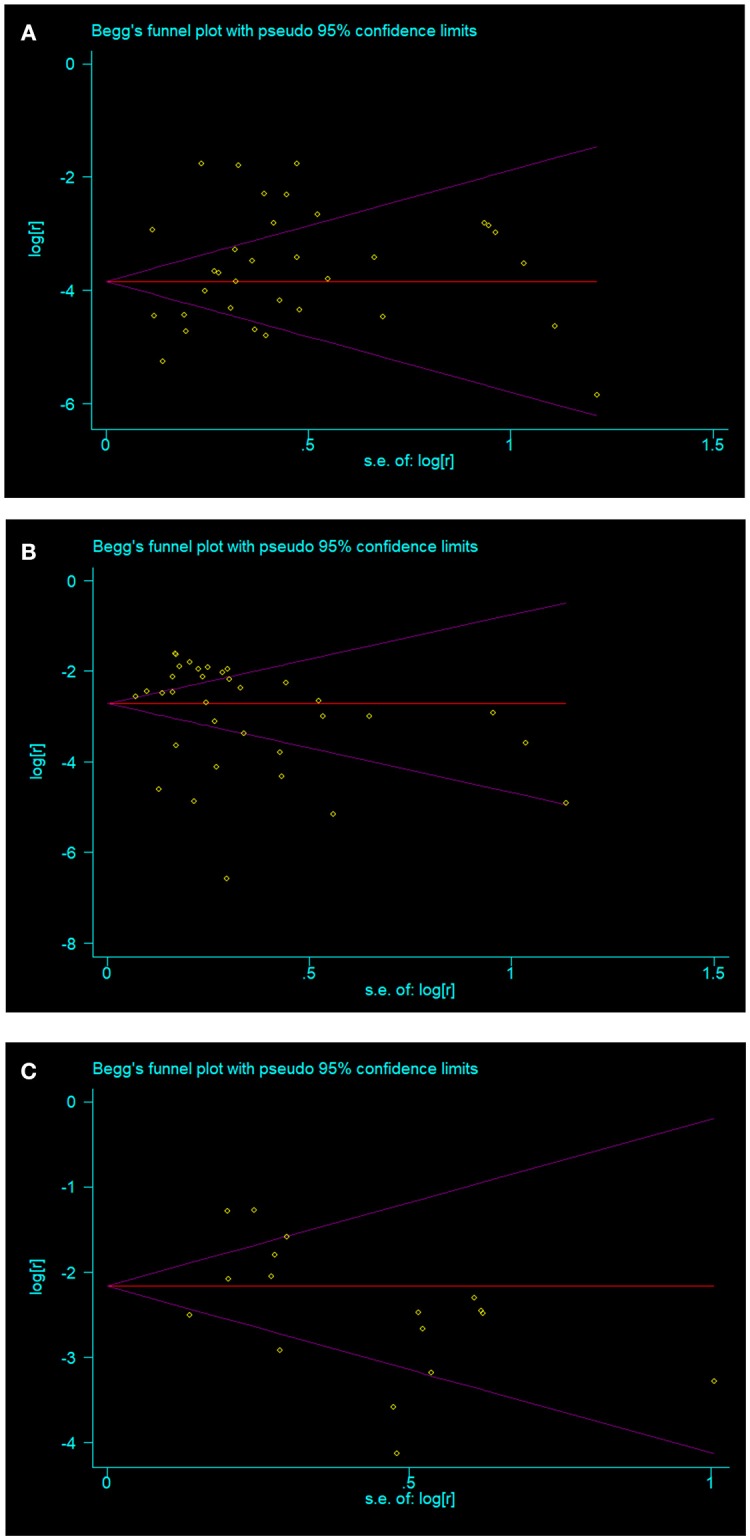
The three thyroid disorders in vitiligo patients with no publication bias **(A)** overt hyperthyroidism; **(B)** overt hypothyroidism; **(C)** subclinical hypothyroidism.

No publication bias were found in papers on overt hyperthyroidism (*t* = 1.16, *p* = 0.256) (Figure 3A), overt hypothyroidism (*t* = 0.95, *p* = 0.350) (Figure 3B), and subclinical hypothyroidism (*t* = −1.36, *p* = 0.194) (Figure 3C). Publication bias was found in the prevalence of Graves disease (*t* = 3.32, *p* = 0.021) and Hashimoto thyroiditis (*t* = 2.96, *p* = 0.012) in patients with vitiligo. Publication bias was not done in subclinical hyperthyroidism in vitiligo patients as there were insufficient observations.

## Discussion

Genome-wide association studies suggesting the relationship between vitiligo and thyroid disorders may be explained by the sharing of a subset of susceptibility gene (89–97). For example, genome-wide linkage analysis in families identified an autoimmunity susceptibility locus on chromosome 1 in patients with both vitiligo and Hashimoto's thyroiditis (96–98). In 2012, Vrijmanc et reported a systematic review about the prevalence of abnormal thyroid function test or elevated thyroid antibodies in vitiligo patients, covering 48 studies (99). The study reminds clinicians should be aware of the possibility of thyroid function changes in patients with vitiligo, however, it did not elaborate specific thyroid dysfunction in vitiligo patients. From a different point, the present systematic review summarized the results of the studies which have categorical diagnoses. The present review involving 77 studies with 3,643 vitiligo subjects supports a significant association between vitiligo and at least one thyroid disorders. The thyroid disorders were subclinical hyperthyroidism, overt hyperthyroidism, Graves disease, subclinical hypothyroidism, overt hypothyroidism, Hashimoto thyroiditis. Twenty-five studies reported the prevalence of subclinical hypothyroidism in vitiligo and the prevalence was the highest (6%) among the six thyroid disorders. Subclinical hyperthyroidism or Graves disease had the lowest prevalence (1%) in vitiligo patients, correspondingly, only approximately 10 studies were, respectively reported about these diseases.

A majority of investigators paid attention to overt hypothyroidism (54 studies) and overt hyperthyroidism (50 studies) in vitiligo patients, although the prevalence of these two disorders (3 and 2%) were lower than that of subclinical hypothyroidism. Overt hypothyroidism patients may experience weight gain, hair loss, dry skin, cold intolerance, constipation, muscle aches, or impaired memory (100–102). Overt hyperthyroidism patients may present with irritability, nervousness and heat intolerance (101, 103).

Our study investigated the potentially distorting factors, including area, gender, age, vitiligo type and stage of vitiligo. The prevalence of overt hyperthyroidism, overt hypothyroidism, Graves disease, and Hashimoto thyroiditis in Europe were higher than in Asia, in contrast, the prevalence of subclinical hypothyroidism in Europe were lower than in Asia. Genetic factor and iodine intaking habit may explain the disparity. The risk of thyroid dysfunction in female vitiligo patients is equal or greater than male, suggesting a gender-relationship between thyroid disorders and vitiligo. Men and women have sexual dimorphism of the immune response (104, 105). The British vitiligo guideline suggests that adult vitiligo patients should regularly screen for thyroid disorders. The present systematic review supports this recommendation in adult vitiligo patients with subclinical hyperthyroidism and overt hyperthyroidism. However, as for subclinical hypothyroidism, overt hypothyroidism and Hashimoto thyroiditis, children had higher prevalence than adult.

In the present review, all thyroid disorders were found in NSV, but not in SV. SV is characterized by early involvement of follicular melanocyte reservoir, early age of onset, and rapid stabilization (106), whereas NSV typically evolves over time and associates with thyroid disease frequently (107). Ethnic background may explain the disparity (91, 107). Different clinical subtypes of NSV have been described, including generalized, acrofacial, and vulgaris types. However, very few studies were included, so we can't draw a clear conclusion. As for the subgroup analysis between active vitiligo and stable vitiligo, 2 thyroid dysfunctions (overt hyperthyroidism and subclinical hypothyroidism) were studied but no definite results were found.

Several limitations of this meta-analysis must be considered. As there were insufficient studies, publication biases were not done about subclinical hyperthyroidism, and publication bias was found in Graves disease and Hashimoto thyroiditis. Studies about vitiligo type and stage were scant. This may have influenced confidence intervals and limited the generalizability of findings. Besides, 3 studies were not included due to the language restrictions.

In conclusion, the systematic review and meta-analysis showed that 6 thyroid disorders showed various prevalence in vitiligo. The highest prevalence was in subclinical hypothyroidism, and the lowest was in subclinical hyperthyroidism or Graves disease. The results of the current review provide useful estimates of the burden of thyroid disorders in vitiligo patients. Screening vitiligo patients for thyroid disorders seem reasonable, in an effort to detect potential thyroid diseases or to assess the risk of future onset.

## Author Contributions

JY and CS conceived, designed and performed the article. SJ, YL, and YZ acquisition of data. H-DC, X-HG, and YW participated in revising the manuscript. All authors contributed to manuscript revision, read and approved the submitted version.

### Conflict of Interest Statement

The authors declare that the research was conducted in the absence of any commercial or financial relationships that could be construed as a potential conflict of interest.
